# Strengthening Mechanism of Char in Thermal Reduction Process of Silicon Dioxide

**DOI:** 10.3390/ma18153651

**Published:** 2025-08-03

**Authors:** Xiuli Xu, Peng Yu, Jinxiao Dou, Jianglong Yu

**Affiliations:** 1 Key Laboratory of Advanced Coal and Coking Technology of Liaoning Province, School of Chemical Engineering, University of Science and Technology Liaoning, Anshan 114051, China; xxl10119@sina.com (X.X.); 24210856020723@stu.ustl.edu.cn (P.Y.); 2 Sinosteel Anshan Research Institute of Thermo-Energy Co., Ltd., Anshan 114044, China; 3 Suzhou Industrial Park Monash Research Institute of Science and Technology, Southeast University-Monash University Joint Graduate School, Suzhou 215123, China

**Keywords:** char, ferrosilicon smelting, reactivity, solid products

## Abstract

This study investigates the strengthening mechanisms of char in silicon dioxide thermal reduction through systematic high-temperature experiments using three char types (YQ1, CW1, HY1) characterized by X-ray diffraction, Raman spectroscopy, transmission electron microscopy, and scanning electron microscopy. HY1 char demonstrated superior reactivity due to its highly ordered microcrystalline structure, characterized by the largest aromatic cluster size (L_a_) and lowest defect ratio (I_D_/I_G_ = 0.37), which directly correlated with enhanced reaction completeness. The carbon–silicon reaction reactivity increased progressively with temperature, achieving optimal performance at 1550 °C. Addition of Fe and Fe_2_O_3_ significantly accelerated the reduction process, with Fe_2_O_3_ exhibiting superior catalytic performance by reducing activation energy and optimizing reaction kinetics. The ferrosilicon formation mechanism proceeds through a two-stage pathway: initial char-SiO_2_ reaction producing SiC and CO, followed by SiC–iron interaction generating FeSi, which catalytically promotes further reduction. These findings establish critical structure–performance relationships for char selection in industrial silicon production, where microcrystalline ordering emerges as the primary performance determinant. The identification of optimal temperature and additive conditions provides practical pathways to enhance energy efficiency and product quality in silicon metallurgy, enabling informed raw material selection and process optimization to reduce energy consumption and improve operational stability.

## 1. Introduction

In recent years, solar and photovoltaic enterprises have developed rapidly, with most solar cells made from silicon. Industrial silicon production typically relies on high-temperature reduction of silica, where the selection of carbon materials critically affects energy consumption, operational stability, and ultimately the yield and quality of the final silicon product [1,2]. Currently, the market demands increasingly stringent quality requirements for silicon [3], making the production process crucial, particularly regarding carbon reducing agents.

Char has emerged as the predominant carbon reducing agent for silicon production due to its superior properties including low ash content, reduced aluminum and phosphorus contents, high electrical resistivity, and strong reducing performance, effectively replacing traditional metallurgical and gas coke. However, significant challenges persist in silicon production, including excessive energy consumption, environmental pollution, and raw material depletion, which have intensified with growing societal demand for silicon.

To address these challenges, extensive research has focused on optimizing carbon–silicon smelting processes. Zhou et al. [4] demonstrated that microwave-treated K_2_CO_3_ and Na_2_CO_3_ additives significantly enhanced carbon–silicon reduction, increasing yields by 54% and 42%, respectively. Technol et al. [5] investigated the effect of Norwegian spruce biochar produced at various temperatures (500, 800, and 1100 °C) mixed with pyrolysis oil and lignosulfonate, finding that additives improved particle density, durability, and reactivity, with optimal performance at 1100 °C. Mekhtiev et al. [6] explored various carbon reducing agents for high-temperature crystalline silicon production, concluding that low-ash special coke and long-flame char were most suitable for silicon smelting reduction.

Temperature effects have also been extensively studied. Bai et al. [7] examined silicon production using char as a reducing agent at 1800, 1900, and 2000 °C, finding optimal results at 2000 °C, confirming that higher temperatures favor carbon–silicon reactions. Ramos et al. [8] conducted extensive reactivity studies of SiO_2_ with different carbon sources, revealing that char reactivity with SiO_2_ increased with decreasing particle size at constant peak carbonization temperature.

Despite these advances, critical knowledge gaps persist regarding the fundamental relationship between char microstructural properties and carbon–silicon reduction performance. While previous studies have optimized reaction conditions and explored various additives, the influence of specific char characteristics, including microcrystalline arrangement, defect density, and structural orderliness, on reaction mechanisms and efficiency remains poorly understood. Moreover, comparative analysis of different char types under identical conditions has been limited, hindering the development of structure–performance relationships essential for rational char selection.

This study addresses these gaps by systematically investigating the strengthening mechanism of char in silicon dioxide thermal reduction through characterization of three char types. Advanced analytical techniques including transmission electron microscopy (TEM), scanning electron microscopy (SEM), Raman spectroscopy, and X-ray diffraction (XRD) establish clear relationships between char microstructural properties and ferrosilicon smelting reactivity. Additionally, we explore Fe and Fe_2_O_3_ catalytic effects and elucidate reaction mechanisms via gas chromatography analysis. This approach provides fundamental insights into char behavior and practical guidance for industrial process optimization through informed reducing agent selection.

## 2. Samples and Experimental

### 2.1. Raw Materials

Three distinct char samples were selected to investigate the influence of different char types on carbon–silicon reduction reactions: Yaoqu Char (YQ1), Chuangwei Char (CW1), and Hongyan Char (HY1). The char samples were placed in self-sealing bags, emptied and sealed, and then stored in a refrigerator to prevent oxidation of the char samples. Prior to each experiment, samples were removed from refrigeration, air-dried under natural sunlight, crushed and ground to below 0.2 mm particle size, and then stored in sealed bags within a desiccator.

High-purity silica (SiO_2_) powder was used as the primary reactant, whilst reduced iron powder (Fe) and iron oxide (Fe_2_O_3_) served as catalytic additives in selected experiments to evaluate their influence on reaction kinetics and product formation.

### 2.2. Analytical Methods

#### 2.2.1. Proximate and Ultimate Analysis

Proximate analysis determined ash content (A) and volatile matter (V) using a well-type muffle furnace (JJR1600-20, Shenyang, China). These parameters provide fundamental indicators for evaluating char quality characteristics and suitability for thermochemical applications.

Ultimate analysis was conducted using an EA3000 automatic elemental composition analyzer (EURO VECTOR, Pavia, Italy) to quantify carbon, hydrogen, nitrogen, sulfur, and oxygen content (calculated by difference). This analysis is essential for understanding char metamorphism degree, calculating calorific values, and estimating pyrolysis product yields.

#### 2.2.2. Reactivity Testing

Char reactivity was assessed using 200 g samples reacted with CO_2_ at 1100 °C for 2 h, with reactivity expressed as the percentage mass loss during this standardized test. This provides a comparative measure of char activity under controlled conditions.

### 2.3. Experimental Apparatus and Procedure

Carbon–silicon smelting experiments were conducted in a custom-built high-temperature reaction furnace equipped with precise thermocouple-based temperature monitoring and control. Because the reaction temperature of the carbon silicon reaction is around 1500 °C, 1500 °C is chosen as the reaction temperature, employing a multi-stage heating protocol that ramped from 25 °C to 200 °C at 6 °C/min, subsequently to 700 °C at 10 °C/min, and finally to 1500 °C at 6.7 °C/min, followed by isothermal holding at the target temperature for 180 min under a high-purity argon protective atmosphere maintained at 100 mL/min flow rate. Typical experiments employed 1.5 g char and 2.9 g SiO_2_, with optional addition of 0.5 g catalytic materials (Fe or Fe_2_O_3_), whilst the reactor design incorporated an integrated gas collection system that enabled simultaneous collection of gaseous products during reaction and recovery of solid products post experiment for comprehensive characterization, thus facilitating complete analysis of both reaction kinetics and product formation mechanisms.

Using a high-temperature resistance furnace for heating from 25 °C to 1500 °C, mass loss of samples was calculated by using an electronic balance and Ar gas as a protective gas during the experiment.

### 2.4. Characterization Techniques

X-ray Diffraction (XRD): Crystalline structure analysis was performed using a Rigaku D/max-2200/PC diffractometer (Rigaku Ultima IV, Tokyo, Japan)with Cu Kα radiation (λ = 0.1541 nm) scanning from 10° to 90° at 2°/min. Peak fitting analysis employed relevant software to extract structural parameters including aromatic interlayer spacing (d_002_), aromatic cluster dimensions (L_c_, L_a_), and graphitization indices.

Raman Spectroscopy: Molecular structural analysis utilized a LabRAM HR confocal Raman spectrometer (HORIBA Jobin Yvon, Palaiseau, France) with 532 nm laser excitation. Samples were demineralized and ground to >200 mesh prior to analysis. Spectral deconvolution identified characteristic bands related to graphitic (G) and defect (D) structures [9,10], enabling calculation of structural ordering parameters.

High-Resolution Transmission Electron Microscopy (HRTEM): Microcrystalline structure imaging employed a Talos F200X G2 microscope (FEI Corporation, Singapore) operating at 200 keV. Samples were ultrasonically dispersed in ethanol and deposited on carbon-coated grids. Images at 1.05-million magnification were processed using MATLAB2019b algorithms to extract carbon layer length distribution, curvature, and interlayer spacing information.

Surface Area Analysis: Nitrogen adsorption–desorption isotherms were measured at −196 °C using a KUBO-X1000 analyzer (Beijing, China) following sample degassing at 150 °C under vacuum for 1 h. Specific surface areas were calculated using the Brunauer–Emmett–Teller (BET) model, whilst pore size distributions employed the Barrett–Joyner–Halenda (BJH) method.

Gas Chromatography: Product gas composition was analyzed using an (Agilent 490, Santa Clara, CA, USA) micro gas chromatograph to quantify CO, H_2_, and other gaseous species. Each measurement represents the average of three replicate analyses.

Scanning Electron Microscopy with Energy Dispersive Spectroscopy (SEM-EDS): Morphological analysis and elemental mapping of reaction products by using the scanning electron microscope model Axia (Thermo Fisher Scientific, Waltham, MA, USA) were conducted to evaluate product distribution and confirm reaction mechanisms.

Using Origin2019b software for peak fitting, the peak position 2θ, peak width at half maximum, peak area, and other related data can be obtained from the peak fitting graph. The aromatic interlayer spacing d_002_, aromatic cluster size L_c_, L_a_, and other data can be calculated using Scherrer Formulas (1) and (2) and Bragg Formula (3) [11,12].

(1) $$\begin{array}{c} L_{c}=\frac{0.9\lambda }{\beta_{002}\cos\theta_{002}} \end{array}$$

(2) $$\begin{array}{c} L_{a}=\frac{1.84\lambda }{\beta_{100}\cos\theta_{100}} \end{array}$$

(3) $$\begin{array}{c} d_{002}=\frac{\lambda }{2sin\theta } \end{array}$$

(4) $$\begin{array}{c} I_{g}=\frac{A_{002}}{{A_{00}}_{2}+A_{\gamma }}\times 100\% \end{array}$$

(5) $$\begin{array}{c} N=1+\left(\frac{L_{c}}{d_{002}}\right) \end{array}$$

where λ is the X-ray wavelength (λ = 0.154056 nm), β_002_ is the half-height width of the 002 diffraction peak, β_100_ is the half-height width of the diffraction peak of 100, θ_002_ is the diffraction angle corresponding to the maximum value of the 002 diffraction peak, θ_100_ is the diffraction angle corresponding to the maximum value of the diffraction peak, d_002_ is the crystal plane spacing of carbon microcrystalline, Ig is the degree of graphitization, A_002_ and A_100_ represent peak areas, and N represents the number of carbon layers.

## 3. Results and Discussion

### 3.1. Sample Characterization

#### 3.1.1. Proximate and Ultimate Analysis of Three Types of Char

The analytical results for the three char types reveal significant compositional differences that influence their thermal reduction performance (Table 1). HY1 char exhibits the highest volatile matter content (~3%), which effectively enhances reaction system activity through generation of active sites during pyrolysis, promoting improved interfacial contact between reactants and optimizing reaction kinetics. The volatile matter liberation creates a more porous structure that facilitates mass transfer during high-temperature reduction processes [13].

From the ultimate analysis perspective, all three chars demonstrate high carbon content (86–90% dry ash-free basis), indicating their suitability as reducing agents. The relatively low ash content, particularly for CW1 (7.63%) and HY1 (8.32%), minimizes potential interference from mineral matter during silicon production, whilst the low sulfur and phosphorus contents meet metallurgical-grade requirements for high-purity silicon applications.

#### 3.1.2. Reactivity Performance

**Figure 1:**
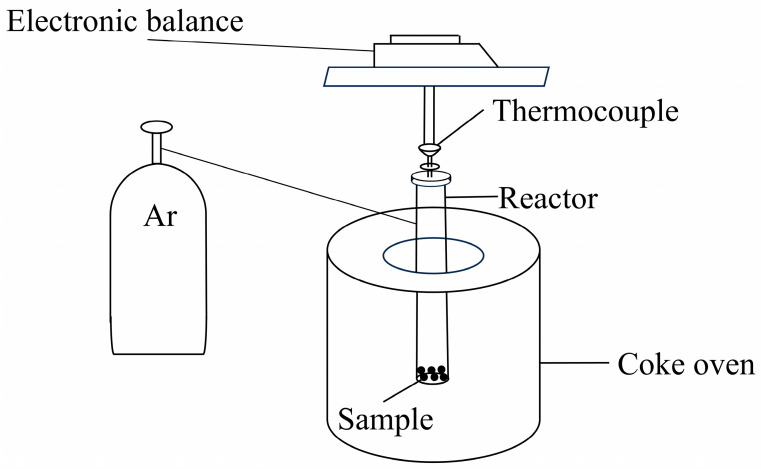
Reaction experimental apparatus.

The reactivity test is the specific mass loss of char and CO_2_ under high temperature conditions of 1100 °C. The temperature-dependent reactivity profiles demonstrate distinct performance characteristics among the three char types (Figure 1). At lower temperatures (750–850 °C), all chars exhibit minimal reactivity, reflecting the activation energy requirements for carbon–CO_2_ gasification. However, as temperature increases beyond 900 °C, significant divergence emerges in their reactive behavior.

**Figure 2:**
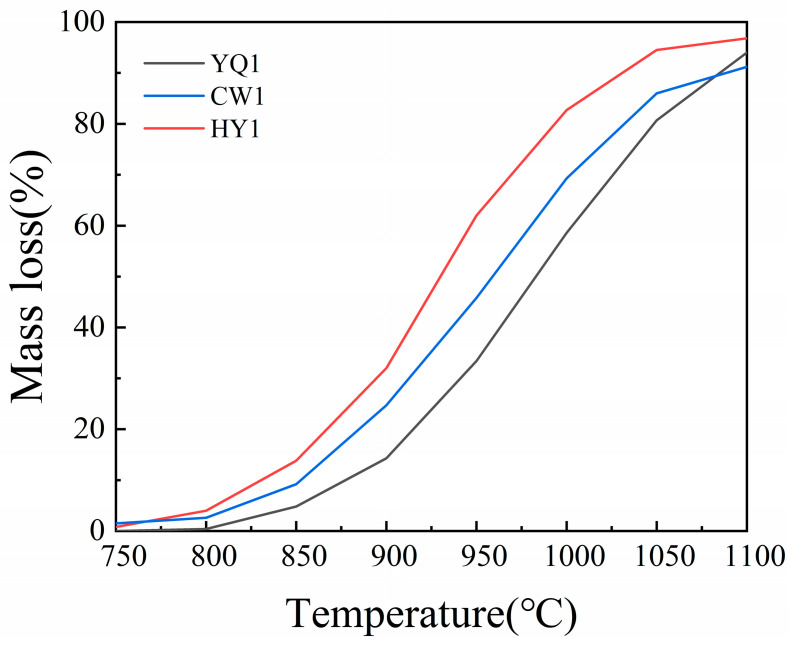
Mass loss (%) of char and metallurgical coke powder of three char types.

HY1 char consistently demonstrates superior reactivity across the entire temperature range, achieving the highest conversion rates at all measurement points. This enhanced performance correlates with its elevated volatile matter content and optimized pore structure development during pyrolysis. The superior reactivity of HY1 indicates greater potential for efficient participation in ferrosilicon smelting reactions compared to YQ1 and CW1 chars (Figure 2).

### 3.2. Effect of the Different Types of Char on the Reaction

**Figure 3:**
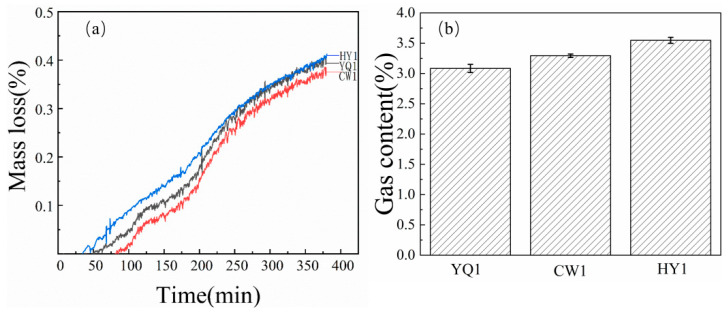
(**a**) Mass loss (%) and (**b**) gas content of SiO_2_ reaction products in char.

Three different systems of 1.5 g and 2.9 g SiO_2_ of char were placed in a crucible for the carbon silicon smelting reaction at 1500 °C. The mass loss of the reaction is shown in Figure 3a. And the gas content, such as CO, H_2_, etc., is shown in Figure 3b.

The reaction weight (%) curve in Figure 3a shows that HY1 exhibited the highest mass loss among the three types of char. And the gas content shown in Figure 3b, it can be concluded that HY1’s gas production is also ahead of the other two types of char in terms of the gas content produced during the reaction, consistent with the results of the mass loss rate. The experimental results indicated that there were significant differences in the carbon thermal reduction reaction between different sources of char under the same reaction conditions. The reactivity between HY1 char and silica was significantly better than that of the other two char materials. Comparative analysis revealed that HY1 char had a higher reactivity index, which could be attributed to its unique pore structure and surface chemical properties. Therefore, from the perspectives of reaction kinetics and thermodynamics, HY1 char was found to be more suitable as a reducing agent for the thermal reduction of silica carbon than the other two types of char.

### 3.3. Microcrystalline Structure Analysis

**Figure 4:**
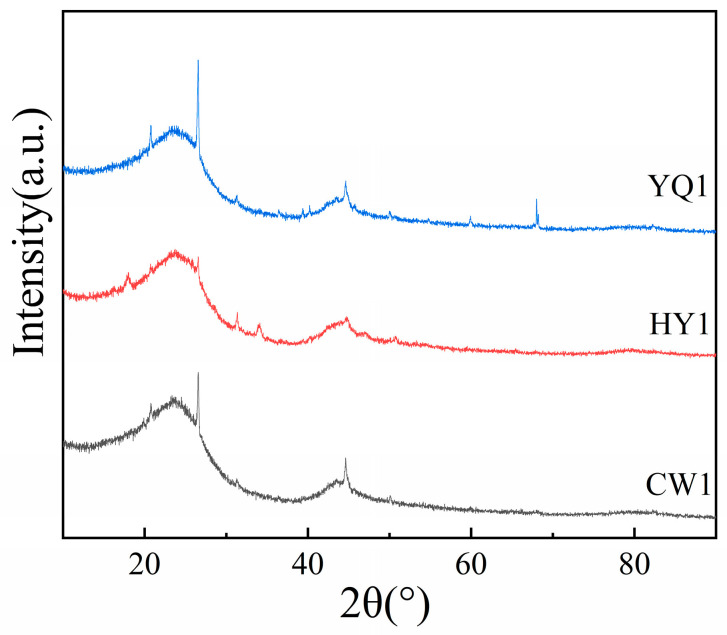
XRD patterns of the three types of char.

X-ray diffraction patterns reveal characteristic features of carbonaceous materials, with distinct gamma peaks at 2θ ≈ 21°, (002) peaks near 2θ ≈ 24°, and (100) peaks at approximately 2θ ≈ 44° (Figure 4). The gamma peak reflects the presence of aliphatic side chains and oxygen-containing functional groups within cyclic aromatic structures, whilst the 002 and 100 peaks represent aromatic layer stacking density and aromatic ring polymerization degree, respectively [14].

**Figure 5:**
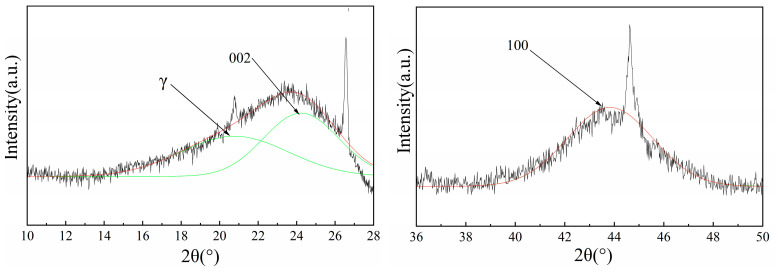
XRD peak fitting spectrum of HY1 char.

Peak fitting analysis (Figure 5) enables quantitative assessment of structural parameters using established mathematical relationships. The calculations reveal that HY1 char possesses the largest aromatic cluster dimensions (L_a_) and highest stacking height (L_c_), indicating a more ordered microcrystalline arrangement [11]. This enhanced structural organization contributes to improved electron transfer capabilities and thermal stability during high-temperature reduction processes [15].

The aromatic interlayer spacing (d_002_) values demonstrate that HY1 maintains optimal spacing for reactant accessibility whilst preserving structural integrity. The higher graphitization index (I_g_) for HY1 confirms its superior structural ordering, which facilitates more efficient carbon–silicon reduction reactions through enhanced electronic conductivity and reduced activation barriers.

**Figure 6:**
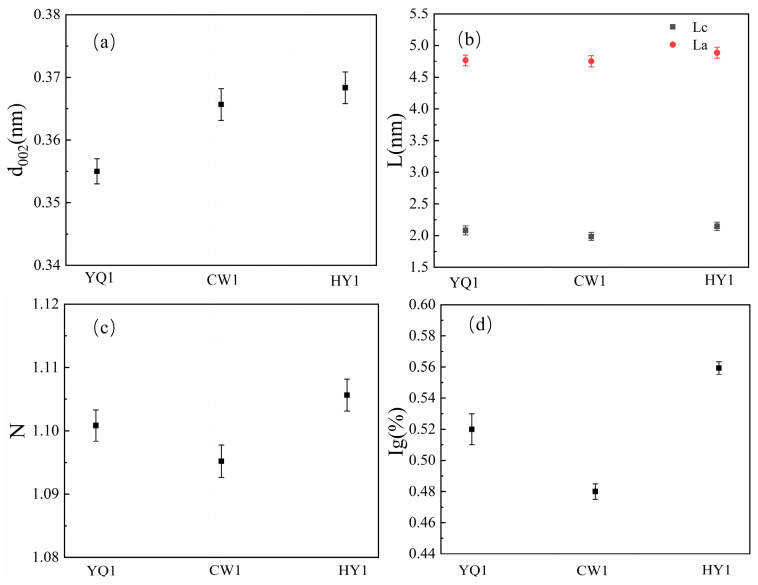
Carbon structure of char. (**a**) d_002_ with different half focal lengths, (**b**) different half focal lengths L_c_ and L_a_, (**c**) different half focal lengths N, and (**d**) different half focal lengths Ig.

During thermal reduction, complex chemical reactions including aliphatic structure fracture and aromatic ring condensation transform amorphous carbon into graphite-like structures (Figure 6). Figure 6a shows that HY1 char exhibits the highest interplanar spacing between microcrystals among the three examined types. This optimal structural spacing facilitates reactant access while maintaining structural integrity, providing an ideal balance for high-temperature reduction processes. Furthermore, the analysis of crystallite dimensions reveals that HY1 demonstrates both the highest stacking height (L_c_) and relatively large lateral size (L_a_) of carbon microcrystals (Figure 6b). This enhanced dimensional ordering directly correlates with improved reactivity, as larger crystallite dimensions typically indicate more developed graphitic structures [16,17].

The carbon layer stacking analysis (Figure 6c) confirms that HY1 char possesses the most extensive carbon layer organization among the three char types, which is entirely consistent with the observed trends in ideal graphite content and the d_002_ spacing between aromatic layers. The superior La content in HY1 char can be attributed to its higher concentration of large aromatic structures, which, combined with its elevated d_002_ and directional structure content, results in the enhanced stacking height (Lc) values observed.

The graphitization degree and Ig content of HY1 are consistently the highest among all tested samples, providing strong evidence that this structural configuration leads to more thorough carbon–silicon reduction reactions. The superior microcrystalline ordering in HY1 creates optimal conditions for electron transfer and thermal conductivity, both essential characteristics for efficient high-temperature reduction processes.

These structural analyses validate that HY1 possesses the most ordered microcrystalline arrangement among the three char types. The enhanced organization manifests through larger aromatic cluster dimensions (L_a_) facilitating improved interfacial contact, increased stacking heights (L_c_) providing thermal stability and electron transfer pathways, optimal interlayer spacing (d_002_) balancing reactant accessibility with structural integrity, and superior graphitization indices (Ig) indicating reduced defect density.

### 3.4. Raman Result of Char

**Figure 7:**
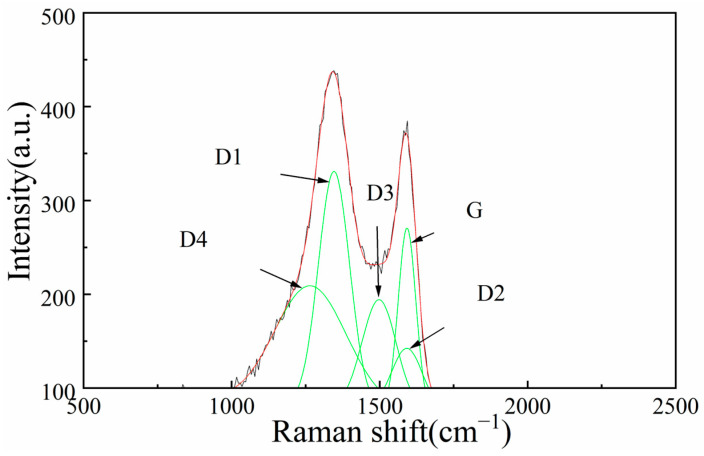
Raman peak fitting spectra of HY1 char.

Spectral deconvolution identifies five characteristic bands following peak fitting procedures (Figure 7). D1 peaks are attributed to heteroatoms on carbon crystal surfaces, D2 peaks represent structural defects in carbon microcrystals, D3 peaks indicate amorphous carbon presence, D4 peaks are from multiple olefin structures, and G peaks represent ideal graphitic crystalline regions [18].

**Figure 8:**
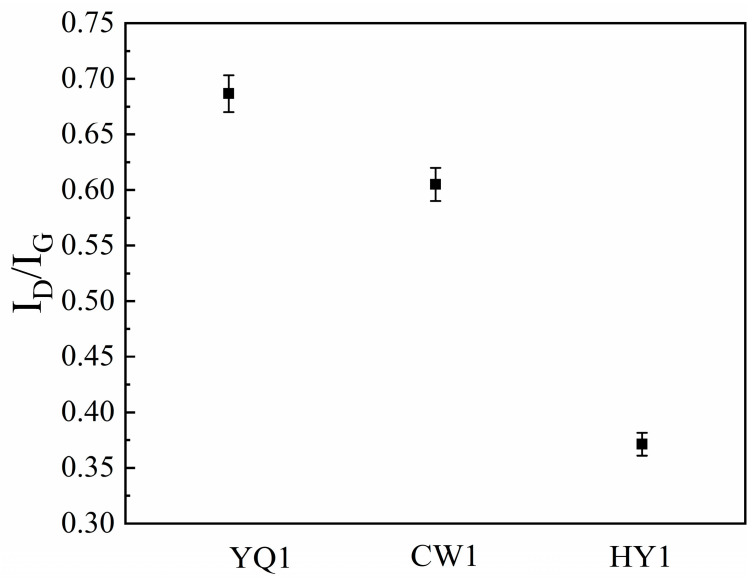
I_D_/I_G_ ratio of the three types of char.

The intensity ratio I_D_/I_G_ serves as an important parameter for assessing structural defect density and crystalline ordering. This ratio provides direct insight into char quality, where lower values indicate microcrystalline arrangement and enhanced reactivity potential. The lower the ratio of I_D_/I_G_, the fewer microcrystal defects and denser the arrangement inside the blue charcoal. Among the three char types we examined, HY1 char exhibits the lowest I_D_/I_G_ ratio (0.37), indicating minimal internal microcrystalline defects (for example: in-plane incompleteness of carbon microcrystals, irregular structural arrangement of carbon microcrystals, etc.), elevated ideal graphite content, and superior reactivity potential (Figure 8) [19]. This reduced defect density correlates directly with the enhanced orderliness observed in XRD analysis and confirms the high-quality microcrystalline structure within HY1 char.

### 3.5. TEM Result of Char

High-resolution transmission electron microscopy provides atomic-level insight into carbon layer organization and arrangement patterns. Computer-assisted image processing enables extraction of lattice fringe information that would otherwise remain obscured in raw micrographs [20].

**Figure 9:**
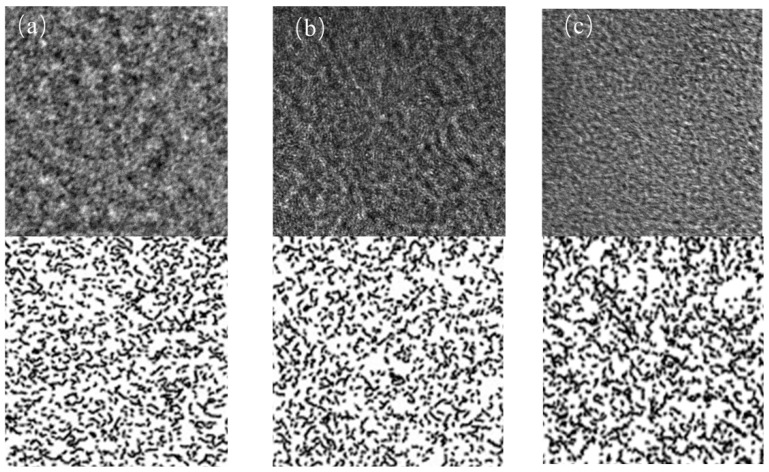
TEM image of char: (**a**) HY1, (**b**) CW1, and (**c**) YQ1.

Figure 9 presents the processed HRTEM images revealing distinct differences in carbon layer organization that correlate strongly with observed reactivity variations among the char samples. Lattice fringe tortuosity, defined as the ratio of actual fringe length to straight-line distance between endpoints, serves as a quantitative measure of structural distortion, with lower values indicating more ordered carbon layers that facilitate electron transport and enhance reaction kinetics [21].

**Figure 10:**
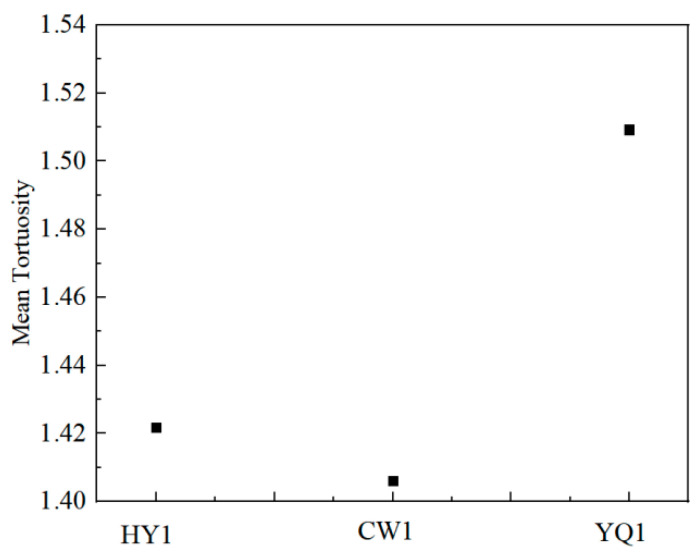
Mean tortuosity of different char.

The processed images in Figure 9 reveal distinct differences in lattice fringe tortuosity among the three char types, as shown in Figure 10. HY1 char demonstrates the lowest mean tortuosity value, indicating highly ordered internal grain structure with minimal structural distortion. This reduced tortuosity correlates directly with improved orderliness and enhanced reactivity, consistent with previous characterization results.

The regular arrangement of carbon microcrystals within HY1 contributes to superior quality and reactivity compared to the more disordered structures observed in YQ1 and CW1 chars. This microscopic evidence supports the macroscopic performance differences observed in mass loss experiments and reinforces HY1’s suitability for efficient thermal reduction applications.

### 3.6. Surface Area and Porosity

**Figure 11:**
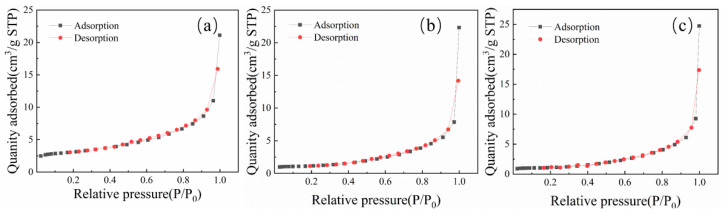
BET test results of three types of char: (**a**) HY1, (**b**)YQ1, and (**c**) CW1.

Nitrogen adsorption–desorption analysis reveals significant differences in pore structure characteristics among the three char types. HY1 exhibits substantially higher specific surface area (10.65 m^2^/g) compared to CW1 (3.71 m^2^/g) and YQ1 (3.85 m^2^/g), indicating a more developed porous network that enhances reactant accessibility and mass transfer rates during thermal reduction. The adsorption curve of HY1 shows a higher starting point and later inflection point (Table 2 and Figure 11), confirming superior pore development and internal surface area availability [22].

The correlation between surface area and microcrystalline structure is noteworthy. HY1 maintains the most ordered carbon arrangement (highest graphitization index, lowest defect ratio) whilst providing the largest accessible surface area. This suggests enhanced reactivity stems from both structural quality and physical accessibility, as ordered microcrystalline domains facilitate electron transfer whilst developed pore structure ensures adequate mass transport.

### 3.7. Analysis of Smelting Results and Products

**Figure 12:**
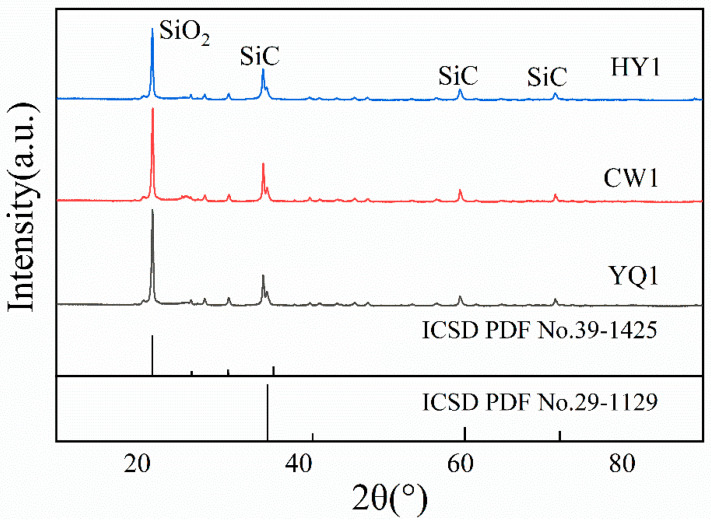
XRD spectra of the reaction products of char with SiO_2_.

XRD characterization of ground reaction products reveals distinct phase compositions that directly correlate with char performance in silicon dioxide thermal reduction (Figure 12). SiC peaks appear at 2θ = 36°, 61°, and 72° [23,24], whilst residual SiO_2_ peaks at 2θ = 23° provide quantitative evidence of reaction completeness. Peak intensity analysis demonstrates significant conversion efficiency differences among char types, with relative intensity ratios of HY1:YQ1:CW1 of approximately 1.307:1.173:1 (Table 3).

HY1 exhibits the lowest residual SiO_2_ content and highest SiC formation. The SiC peak at 2θ = 61° shows markedly higher intensity for HY1, confirming more complete carbon–silicon interaction. This superior performance stems from the unique combination of structural ordering (largest L_a_, highest L_c_, minimal I_D_/I_G_ ratio of 0.37) and enhanced surface accessibility demonstrated earlier, which promotes efficient mass transport whilst maintaining reactive site availability.

Quantitative analysis reveals systematic reduction in unreacted SiO_2_ following HY1 > YQ1 > CW1 (Table 3), mirroring reactivity trends from mass loss and gas evolution studies. The XRD patterns indicate that reduction proceeds through intermediate SiC formation rather than direct silicon production at 1500 °C, where SiC formation is thermodynamically favored, due to the fact that the ∆G value for producing SiC at this temperature is greater than 0; the value is 130 kJ/mol (1500 °C). The ordered aromatic layers in HY1 provide enhanced electron conductivity, reducing activation barriers for the reduction reaction and enabling more complete conversion under identical reaction conditions.

### 3.8. Effect of Processing Parameters on Char Reactivity

Thermal processing conditions significantly influence char structural evolution and subsequent reactivity in silicon reduction reactions. To investigate this relationship, HY char was prepared under two distinct heating protocols: HY1 employed a controlled heating rate of 5 °C/min whilst HY2 utilized an accelerated 8 °C/min profile, both reaching 1100 °C. These processing parameters directly affect the development of microcrystalline structure, volatile matter evolution, and pore formation mechanisms during carbonization.

**Figure 13:**
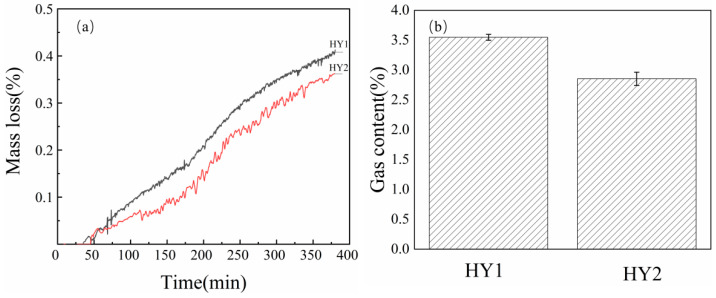
(**a**) Mass loss (%) and (**b**) gas content of reaction products for HY char at different heating rate.

Mass loss analysis (Figure 13a) demonstrates that HY1 char achieves superior conversion efficiency compared to HY2, with final mass loss rates of approximately 0.42% versus 0.38%, respectively. This enhanced reactivity correlates with the more controlled structural development achieved through slower heating rates, which facilitates optimal aromatic layer organization and reduces defect formation. Gas chromatography analysis (Figure 13b) confirms this trend, showing significantly higher CO and H_2_ generation for HY1 char, indicating more complete participation in the reduction reaction.

### 3.9. The Effect of Additives on the Reaction

In recent years, petroleum coke, carbonaceous waste, and other additives were used to study the carbon silicon reaction. The conclusion can be drawn that material substitution can be used to improve alloy quality by reducing impurity content [25,26]. In this work, given the high-temperature nature of char reduction reactions and the potential for iron–silicon compound formation [27], Fe and Fe_2_O_3_ were selected as catalytic additives to evaluate their influence on reaction kinetics and product formation.

#### 3.9.1. Iron Powder Addition

**Figure 14:**
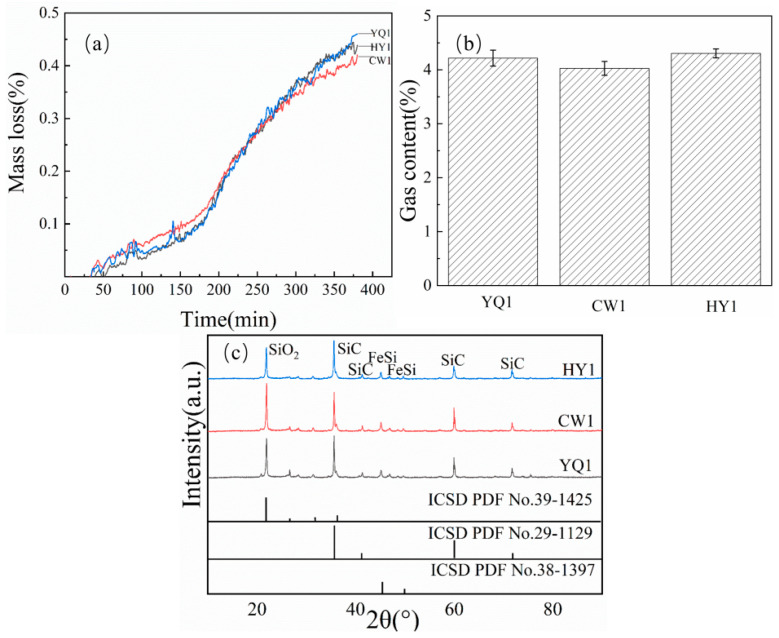
(**a**) Mass loss (%), (**b**) gas content, and (**c**) XRD patterns of reaction products with addition of Fe.

Carbon thermal reduction experiments using char, SiO_2_, and reduced iron powder revealed significant catalytic effects (Figure 14). TGA results demonstrated that HY1 char exhibited substantially enhanced mass loss compared to YQ1 and CW1 after Fe addition. The mass loss of HY1 increased from 1.75 g to 2.25 g (28.6% enhancement), whilst the other char types showed similar improvement trends, confirming the positive catalytic effect of Fe on carbon–silicon reduction (Figure 3a and Figure 14a).

Gas evolution analysis (Figure 14b) showed HY1 maintained the highest gas production after iron addition, consistent with mass loss trends and indicating enhanced thermodynamic driving force. XRD characterization of reaction products (Figure 14c) revealed distinct phase formation: SiC peaks at 2θ = 36°, 61°, and 72°; residual SiO_2_ at 2θ = 23°; and FeSi peaks at 2θ = 44° and 52°, confirming ferrosilicon formation. Notably, HY1 exhibited the weakest SiO_2_ peak intensity, indicating superior conversion efficiency.

#### 3.9.2. Iron Oxide Addition

**Figure 15:**
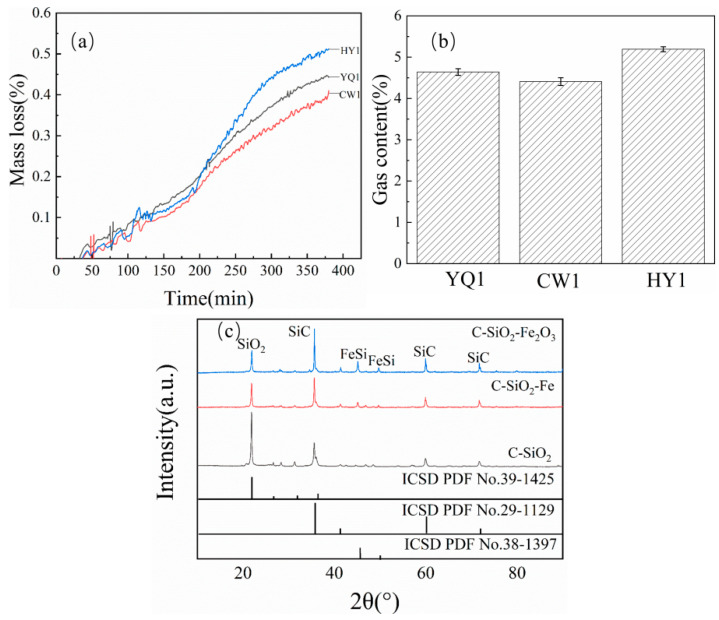
(**a**) Mass loss (%), (**b**) gas content, and (**c**) XRD patterns of reaction products with addition of Fe_2_O_3_.

Fe_2_O_3_ addition produced even more pronounced catalytic effects than metallic iron. HY1 mass loss increased from 1.75 g to 2.5 g, representing a greater enhancement than observed with Fe powder (Figure 15a). Gas production analysis confirmed this superior performance, with HY1 maintaining the highest yields across all experimental conditions (Figure 15b).

XRD analysis revealed that Fe_2_O_3_ promoted more complete SiO_2_ conversion and enhanced FeSi formation compared to metallic iron addition, with notably higher FeSi peak intensities indicating superior catalytic performance through three synergistic mechanisms: the metallic iron generated from Fe_2_O_3_ reduction decreases reaction activation barriers, Fe/FeO phase formation promotes interfacial mass transport, and iron phase presence optimizes reaction system microstructure, collectively creating a more efficient reduction environment that facilitates both carbon–silicon interactions and ferrosilicon product formation [28].

#### 3.9.3. Comparative Catalyst Performance

The experimental results confirm that the addition of Fe and Fe_2_O_3_ significantly promoted the progress of the carbon thermal reduction reaction. The mass loss analysis data showed that the experimental group with added Fe and Fe_2_O_3_ had significantly increased mass loss, and the amount of generated gas-phase products also increased. The XRD pattern analysis confirmed this conclusion. These data indicate that the introduction of Fe and Fe_2_O_3_ significantly improves the conversion rate of SiO_2_ and generation of SiC. Comparing the effects of the two catalysts, Fe_2_O_3_ exhibited superior catalytic performance.

### 3.10. Effect of Reaction Temperature on Ferrosilicon Smelting Performance

Temperature significantly affects carbon–silicon reduction kinetics and equilibrium composition in ferrosilicon smelting. To systematically investigate temperature effects on carbon–silicon reduction performance, experiments were conducted using optimized reaction mixtures comprising 1.5 g HY1 char, 0.5 g reduced iron powder, and 2.9 g SiO_2_ at isothermal holding temperatures of 1450 °C and 1550 °C, with comparative analysis against the baseline 1500 °C condition.

**Figure 16:**
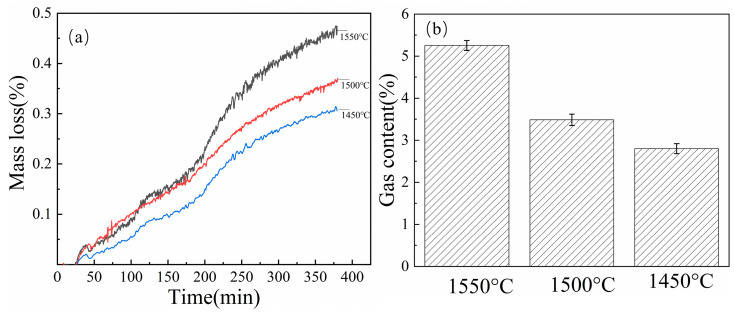
(**a**) Mass loss (%) and (**b**) gas content of reaction products at different temperatures for HY1 char with SiO_2_ and Fe.

Figure 16a shows pronounced temperature sensitivity in mass loss profiles, with higher temperatures promoting higher conversion efficiency. At 1550 °C, maximum mass loss reached 45%, compared to 38% at 1500 °C and 32% at 1450 °C, representing increases of 18.4% and 40.6%, respectively. This temperature dependence reflects the endothermic nature of carbothermic reduction and exponential Arrhenius relationships governing reaction kinetics.

Corresponding gas evolution data (Figure 16b) reveals systematic CO production increases with temperature, confirming enhanced carbon consumption. At 1550 °C, gas generation reached 5.8%, substantially exceeding 3.7% at 1450 °C, which is a 56.8% increase indicating more complete carbon participation in reduction reactions.

Enhanced performance at higher temperatures stems from increased carbon activation through surface atom mobility and reduced activation barriers for carbon–oxygen bond formation. Temperature also affects competing equilibria between SiC and metallic silicon formation, with higher temperatures favoring silicon production essential for ferrosilicon synthesis.

The results establish 1550 °C as optimal within the investigated range, balancing reaction completeness against energy consumption. Temperature variations of 50 °C cause substantial changes in conversion efficiency, validating the importance of precise thermal control in industrial ferrosilicon production. The enhanced catalytic effect of Fe additives becomes more pronounced at elevated temperatures, suggesting synergistic thermal–catalytic interactions warrant further investigation for process optimization.

## 4. Reaction Mechanism

The systematic characterization reveals fundamental mechanisms governing char-enhanced SiO_2_ thermal reduction. HY1 char demonstrates superior performance through its highly ordered microcrystalline architecture characterized by the largest aromatic cluster size (L_a_ = 4.8 nm) and lowest defect density (I_D_/I_G_ = 0.37), which creates optimal electron transfer pathways that reduce activation barriers for carbon–silicon interactions. This enhanced electronic conductivity, combined with superior surface area (10.65 m^2^/g), establishes synergistic effects between structural ordering and porosity development that minimize mass transport limitations whilst maximizing reactive site density, evidenced by 28.6% increased mass loss compared to other char types.

**Figure 17:**
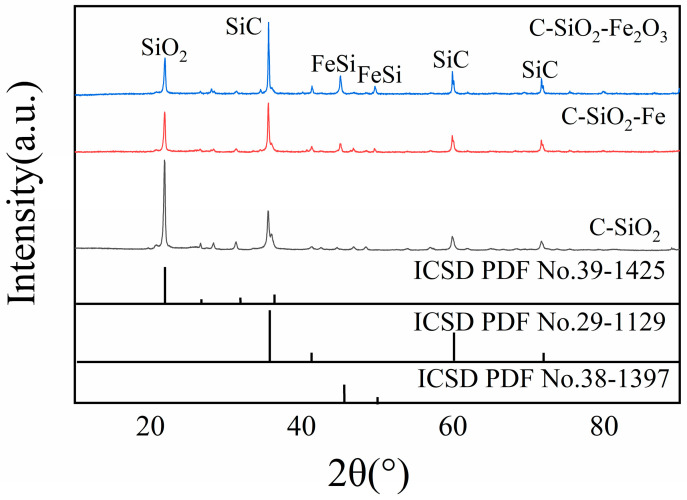
Comparison of XRD spectra of reaction products under different conditions.

The primary reduction pathway proceeds through silicon carbide formation according to SiO_2_(s) + 3C(s) → SiC(s) + 2CO(g), with XRD analysis confirming SiC peaks at 2θ = 36°, 61°, and 72° as the dominant thermodynamically favorable route [29,30,31]. Gas chromatographic analysis revealing CO as the predominant product supports this mechanism, whilst the reaction proceeds through distinct temporal stages: initial interface formation, primary SiC formation with maximum CO evolution, and conversion completion through equilibrium establishment. XRD comparison between original and additive-containing samples (Figure 17) clearly demonstrates enhanced SiO_2_ participation and increased SiC product formation, confirming that iron and iron oxide additions promote the forward reaction through thermodynamic mechanisms.

Fe alters reduction kinetics by providing alternative pathways that circumvent activation energy barriers through ferrosilicon formation via Fe(s) + SiC(s) → FeSi(s) + C(s) and Fe(l) + Si(l) → FeSi(l), creating thermodynamically favorable sinks confirmed by XRD peaks at 2θ = 44° and 52°.

**Figure 18:**
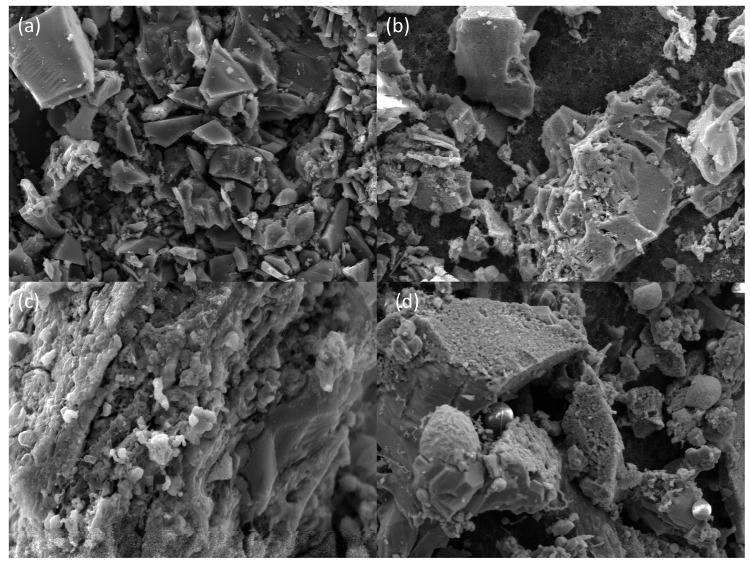
Electron microscopy image of the reaction product between char and silicon: (**a**) C, (**b**) C-SiO_2_, (**c**) C-SiO_2_-Fe, and (**d**) C-SiO_2_-Fe_2_O_3_.

SEM analysis of HY1 char structure (Figure 18a) reveals block-like morphology with smooth surfaces that maintain mechanical strength whilst preventing structural collapse, whilst reaction products (Figure 18b–d) demonstrate char corrosion and Fe compound formation indicating successful FeSi generation.

**Figure 19:**
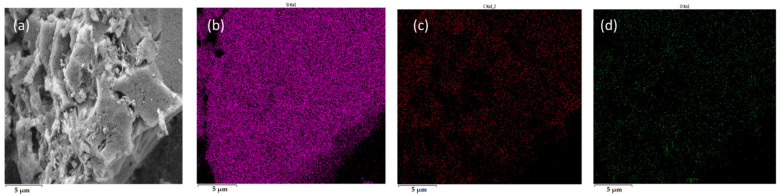
SEM image of the reaction between C and SiO_2_: (**a**) electron microscopy image of the product, (**b**) Si, (**c**) C, and (**d**) O.

**Figure 20:**
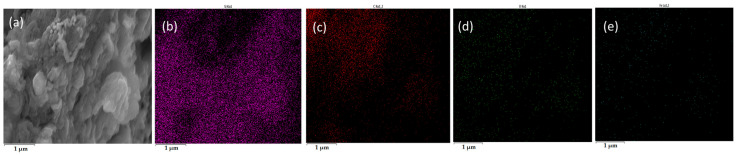
SEM image of the reaction between C, Fe, and SiO_2_. (**a**) Electron microscopy image of the product. (**b**) Si, (**c**) C, (**d**) O, and (**e**) Fe.

SEM elemental mapping of carbon–silicon dioxide reactions (Figure 19) shows uniform distribution of carbon, silicon, and oxygen elements, confirming complete reactant interaction, whilst Fe catalysis mechanisms become evident through char–silica–Fe analysis (Figure 20), where decreased carbon content indicates enhanced char participation due to iron promoting localized high-activity regions.

**Figure 21:**
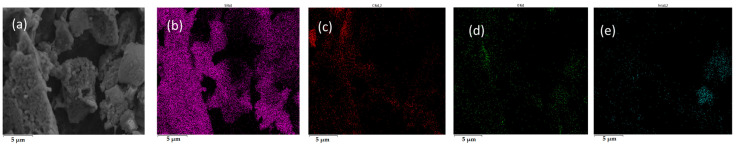
SEM image of the reaction between C, Fe_2_O_3_, and SiO_2_. (**a**) Electron microscopy image of the product, (**b**) Si, (**c**) C, (**d**) O, and (**e**) Fe.

Fe_2_O_3_ exhibits superior catalytic performance through in situ reduction creating highly active nascent iron surfaces according to Fe_2_O_3_(s) + 3C(s) → 2Fe(s) + 3CO(g), with concurrent CO generation providing additional reducing atmosphere. SEM analysis of char–silica–Fe_2_O_3_ reactions (Figure 21) reveals increased oxygen content from Fe_2_O_3_, whilst decreased carbon content confirms enhanced char participation compared to metallic iron addition, establishing synergistic coupling where oxygen release creates oxygen-deficient regions, facilitating SiO_2_ decomposition through altered local thermodynamics. This mechanism explains Fe_2_O_3_’s superior catalytic performance evidenced by higher FeSi peak intensities in XRD analysis, with temperature optimization at 1550 °C enabling kinetic control through enhanced atomic mobility and reduced activation barriers.

## 5. Conclusions

This systematic investigation establishes that microcrystalline ordering, rather than surface area or volatile content, governs carbon–silicon reduction smelting. Through comprehensive structural characterization using XRD, Raman spectroscopy, and electron microscopy, we demonstrate critical structure–performance relationships that enable rational char selection for industrial silicon production.

HY1 char exhibited superior reactivity due to its highly ordered microcrystalline structure, characterized by the largest aromatic cluster dimensions (L_a_ = 4.9 nm), highest stacking height (L_c_ = 2.1 nm), and minimal defect density (I_D_/I_G_ = 0.37). This enhanced structural organization facilitates electron transfer pathways and reduces activation barriers, directly correlating with improved reaction completeness. The ordered aromatic layers provide enhanced electronic conductivity whilst optimal interlayer spacing maintains reactant accessibility without compromising structural integrity.

Fe-based additives significantly accelerated reduction kinetics through catalytic enhancement. Fe_2_O_3_ demonstrated superior performance over metallic Fe, increasing conversion efficiency by 43% via a two-stage mechanism: initial char–SiO_2_ reaction producing SiC and CO, followed by SiC–iron interaction generating ferrosilicon compounds that catalytically promote further reduction. Temperature optimization revealed progressive reactivity enhancement, achieving optimal performance at 1550 °C where both mass loss and gas evolution reached maximum values.

These findings establish quantitative criteria for char selection based on microstructural parameters (L_a_, L_c_, I_D_/I_G_ ratios) rather than conventional proximate analysis. The identified structure–performance relationships provide immediate pathways to enhance energy efficiency and product quality in industrial silicon production whilst supporting development of more sustainable metallurgical processes through reduced energy consumption and improved operational stability.

## Figures and Tables

**Table 1:** Proximate and ultimate analysis of three types of char.

	Items	YQ1	CW1	HY1
Proximate analysis (wt.%, ad)	Ash	11.54	7.63	8.32
	Volatility	2.46	1.76	2.95
Ultimate analysis (wt.%, daf)	C	86.04	90.41	90.00
	H	0.56	0.54	0.50
	O *	0.96	0.40	0
	N	0.71	0.75	0.82
	S	0.20	0.28	0.37

ad: air-dried basis; daf: dry ash-free basis; * calculated by difference.

**Table 2:** Starting point and inflection point of three types of char.

	HY1	YQ1	CW1
Starting point (cm^3^/g STP)	2.49	1.01	0.98
Inflection point (cm^3^/g STP)	8.97	7.21	7.03

**Table 3:** Peak intensity of reactants of three types of char.

Sample	HY1	YQ1	CW1
Peak intensity of SiO_2_ (a.u.)	9293	10,859	12,152

## Data Availability

The original contributions presented in this study are included in the article. Further inquiries can be directed to the corresponding authors.
